# Recent Developments in 1,3-*β*-Glucan Biology:  Proceedings of the 5th Glucan Symposium 
Tokyo, December 8, 2006

**DOI:** 10.1155/2007/79648

**Published:** 2008-01-03

**Authors:** Edited by: Ragnar Rylander

**Affiliations:** BioFact Environmental Health Research Center, Bjorkasv 21, 44391 Lerum, Sweden

The following workshops reported on the advancements on 1,3-*β*-glucan biology in terms of understanding the molecular basis for the cellular reactions induced as well as new methods for analysis and the clinical applications, particularly concerning fungal infections.

The first glucan workshop was organized in 1990 with the purpose to draw attention to the importance of 1,3-*β*-glucan, one of the major constituents of fungal cell walls, as a bioactive agent in our environment [1]. The workshop reviewed available data and through fruitful discussions encouraged further research in the area. This was followed by a succession of similar workshops in 1993, 1994, and 1997 [2–4].

Although the number of researchers in this field has remained fairly limited over the years, progress since the first workshop has been significant. Several subjects at the present workshop are similar to those treated 17 years ago but there has been an imposing increase in depth and breath of the knowledge.

In the first workshop, a main interest was the methodology of the measurement of 1,3-*β*-glucan and the levels present in the environmental and in clinical specimens. Information was also presented on the influence of 1,3-*β*-glucan on inflammatory and immunological systems. It was suggested that 1,3-*β*-glucan could be an important environmental and clinical agent related to human diseases but there was a lack of both experimental and clinical studies. Against this short background of previous workshops it is time again to present the advances in the different fields, now with an emphasis on molecular biology for the understanding of the effects and experience from investigations on humans for clinical and environmental risk evaluations.

## Figures and Tables

**Table 1:** 1,3-*β*-glucan (Limulus lysate analysis) in different environments.

Environment	*n*	Range	References
**Airborne ng/m^3^**			
Poultry farms	39	4.1–870	[3]
Waste collection	14	2.0–34.0	[6]
Waste collection	25	5–220	[7]
Cotton cardroom	15	1 037–7 286	[8]
Mouldy homes	26	0–7.6	[9]
Recycling	136	0–137	[11]
Wood processing	24	4.5–330	[8]
Horse stables	20	0–76.5	[8]
Swine stables	9	78.6–425	[8]
Homes, indoor/outdoor	19	0.3–9.4	[9]

**Airborne agitated ng/m^3^**			
Mouldy homes	26	0–9.3	[10]
School	15	0–27.4	[12]
Row houses	75	0–19.0	[13]
Day care	9	1.4–11.0	[14]
House	6	5–106	[15]

**Sedimented dust ng/mg**			
Mouldy homes	26	1.5–173	[9]
Cotton cardroom	4	572–850	[8]

**Table 2:** 1,3-*β*-glucan (immunological method analysis) in different environments.

Environment	*n*	Range	References
**Airborne *μ*g/m^3^**			
Waste collectors	118	0–30.8	[16]
Saw mills	37	0.4–92.5	[17]

**Sedimented dust**			
Homes (*μ*g/g)	476	98–10 000	[18]
Homes (*μ*g/m^2^)	75	157–3 652	[19]
Homes (*μ*g/m^2^, GM)	211	0–243	[20]
Homes (*μ*g/g, percentiles)	395	469–4 065	[21]
