# Supplementary material for: Recent Developments in 1,3-β-Glucan Biology: : Proceedings of the 5th Glucan Symposium : Tokyo, December 8, 2006
Source: Mediators Inflamm. 2008 Jan 3;2007:79648. doi: 10.1155/2007/79648 (PMC2327214; doi:10.1155/2007/79648)
Supplement: Related texts

# Abstract: INVENTORY OF 1,3-*β*-GLUCAN LEVELS IN THE ENVIRONMENT

**Authors:** Ragnar Rylander

**Affiliations:** BioFact Environmental Health Research Center, Bjorkasv 21, 44391 Lerum, Sweden

## INTRODUCTION

The mould cell wall agent 1,3-*β*-glucan has a number of biological effects after inhalation and injection [1]. The purpose of this presentation is to review data from some major studies on the levels of 1,3-*β*-glucan in occupational and general environments.

## METHODOLOGICAL ISSUES

The amount of 1,3-*β*-glucan in environmental samples can be determined using two different analytical methods. One method is the Limulus assay using a glucan specific lysate [2]. A sample of airborne or sedimented dust is extracted in water with NaOH added or during heating to 130°C. This procedure is required to dissolve the triple helix of the glucan molecule and render it accessible to the reagent in the test. If this treatment is not performed, the analysis will comprise only the water soluble portion of the environmental glucan which is 10% or less than the total [3]. The method is similar to the standard method to analyse bacterial endotoxin where a specific lysate is also used. The method has a high sensitivity (around 1 pg/mL) but is not specific for moulds as pollen and certain vegetable fibres also contain 1,3-*β*-glucan.

Another method to determine that 1,3-*β*-glucan is based on a specific enzyme immunoassay [4]. It uses purified polyclonal rabbit anti-1,3-*β*-glucan antibodies and laminarin (a 1,3-*β*-glucan) as the coated antigen and calibration standard. The detection threshold for this method is 0.26 *μ*g/m^3^. No data comparing the results from the two methods in the same sample have been published.

## ENVIRONMENTAL MEASUREMENTS

### The Limulus method

The first study where the level of 1,3-*β*-glucan was measured in indoor environments comprised a number of flats, daycare centers, and a post-office [5]. After that a number of studies in indoor environments and occupational exposures have been published. The results are summarised in Table 1.

The highest values were recorded in animal confinement buildings. The high levels in cotton cardrooms with a relatively small variation suggest a reaction with the cotton fibre.

### The immunological method

A number of field studies report levels of indoor 1,3-*β*-glucan using the immunological method. They are summarised in Table 2.

### Comments

A review of most of the available data on 1,3-*β*-glucan in different environments demonstrates a variation of dose levels within the different environments. Values using the Limulus method are usually considerably lower illustrating the very high sensitivity of that method.

In several of the studies reviewed above, the levels of 1,3-*β*-glucan have been related to the presence of symptoms or clinical measures of inflammation, either comparing high and low/control exposures or on a dose-response basis. A relation has been found for the extent of respiratory symptoms [11], pulmonary function [19], and blood lymphocyte numbers [6]. Although significant relations have been found, they do not prove causality for 1,3-*β*-glucan as the environments studied contain a variety of biologically active agents such as bacterial endotoxin, fungal toxins, fungal enzymes, and microbial cell wall agents from a variety of different microbes. Further studies are required to assess the role of 1,3-*β*-glucan for these effects. For preventive purposes, it could be possible to use the level of 1,3-*β*-glucan as a descriptor of the exposure and as a basis for “action values”. The available data are not yet sufficient to suggest those action values although 25 ng/mg floor dust has been suggested for an increased risk for high levels of IgE [10]. Further work is required to define such values.

## References

[1] Young S-H, Castranova V (2005). *Toxicology of* 1 → 3 *-Beta-Glucans: Glucans as a Marker for Fungal Exposure*.

[2] Tamura H, Arimoto Y, Tanaka S, Yoshida M, Obayashi T, Kawai T (1994). Automated kinetic assay for endotoxin and in (1 → 3) − *β*-D-glucan human blood. *Clinica Chimica Acta*.

[3] Rylander R, Carvalheiro MF (2006). Airways inflammation among workers in poultry houses. *International Archives of Occupational and Environmental Health*.

[4] Douwes J, Doekes G, Montijn R, Heederik D, Brunekreef B (1996). Measurement of *β*(1 → 3)-glucans in occupational and home environments with an inhibition enzyme immunoassay. *Applied and Environmental Microbiology*.

[5] Rylander R, Persson K, Goto H, Yuasa K, Tanaka S (1992). Airborne beta-1,3-glucan may be related to symptoms in sick buildings. *Indoor and Built Environment*.

[6] Thorn J, Beijer L, Rylander R (1998). Airways inflammation and glucan exposure among household waste collectors. *American Journal of Industrial Medicine*.

[7] Heldal K, Halstensen AS, Thorn J, Eduard W, Halstensen TS (2003). Airway inflammation in waste handlers exposed to bioaerosols assessed by induced sputum. *European Respiratory Journal*.

[8] Rylander R

[9] Lee T, Grinshpun SA, Kim KY, Iossifova Y, Adhikari A, Reponen T (2006). Relationship between indoor and outdoor airborne fungal spores, pollen, and (1 → 3) − *β*-D-glucan in homes without visible mold growth. *Aerobiologia*.

[10] Rylander R, Andersson B Microbial cell wall agents indoors in relation to some markers of inflammation.

[11] Gladding T, Thorn J, Stott D (2003). Organic dust exposure and work-related effects among recycling workers. *American Journal of Industrial Medicine*.

[12] Rylander R, Norrhall M, Engdahl U, Tunsäter A, Holt PG (1998). Airways inflammation, atopy, and (1 → 3) − *β*-D-glucan exposures in two schools. *American Journal of Respiratory and Critical Care Medicine*.

[13] Thorn J, Rylander R (1998). Airways inflammation and glucan in a rowhouse area. *American Journal of Respiratory and Critical Care Medicine*.

[14] Rylander R (1997). Airborne (1 → 3) − *β*-D-glucan and airway disease in a day-care center before and after renovation. *Archives of Environmental Health*.

[15] Rylander R, Hsieh V, Courteheuse C (1994). The first case of sick building syndrome in Switzerland. *Indoor and Built Environment*.

[16] Wouters IM, Hilhorst SKM, Kleppe P (2002). Upper airway inflammation and respiratory symptoms in domestic waste collectors. *Occupational and Environmental Medicine*.

[17] Douwes J, McLean D, van der Maarl E, Heederik D, Pearce N (2000). Worker exposures to airborne dust, endotoxin and *β*(1,3)-glucan in two New Zealand sawmills. *American Journal of Industrial Medicine*.

[18] Schram-Bijkerk D, Doekes G, Boeve M (2005). Bacterial and fungal components in house dust of farm children, Rudolf Steiner school children and reference children: the PARSIFAL study. *Allergy*.

[19] Douwes J, Doekes G, Heinrich J, Koch A, Bischof W, Brunekreef B (1998). Endotoxin and *β* (1 → 3)-glucan in house dust and the relation with home characteristics: a pilot study in 25 German houses. *Indoor Air*.

[20] Douwes J, Zuidhof A, Doekes G (2000). (1 → 3) − *β*-D-glucan and endotoxin in house dust and peak flow variability in children. *American Journal of Respiratory and Critical Care Medicine*.

[21] Gehring U, Douwes J, Doekes G (2001). *β*(1 → 3) -glucan in house dust of German homes: housing characteristics, occupant behavior, and relations with endotoxins, allergens and molds. *Environmental Health Perspectives*.

# Abstract: PULMONARY EXPOSURE TO CANDIDA SOLUBLE CELL WALL 1,3-*β*-GLUCAN INDUCES LUNG INFLAMMATION IN MICE

**Authors:** K. Inoue, T. Oda, R. Yanagisawa, H. Tamura, N. Ohno, Y. Adachi, K. Ishibashi, T. Yoshikawa

**Affiliations:** ^1^Environmental Health Sciences Division, National Institute for Environmental Studies, Tsukuba, Ibaraki 305-8506, Japan; ^2^Inflammation and Immunology, Kyoto Prefectural University of Medicine, Kyoto 602-0841, Japan; ^3^Seikagaku Corporation, Tokyo, Japan; ^4^Laboratory for Immunopharmacology of Microbial Products, School of Pharmacy, Tokyo University of Pharmacy and Life Sciences, 1432-1 Horinouchi, Hachioji, Tokyo 192-0392, Japan

It is widely recognized that cell wall components of microorganisms may induce variety of host reactions. The cell wall component lipopolysaccharide of Gram-negative bacteria and peptidoglycan of Gram-positive bacteria can activate macrophages to release proinflammatory molecules, resulting in circulatory shock and multiple organ failure [1, 2]. On the other hand, the bioactivity of cell wall component(s) of fungi has not been fully elucidated, especially in vivo. In a previous study, we isolated soluble 1,3-*β*-glucan (CSBG) from *Candida albicans* [3]. In the current work, we investigated the effects of CSBG on the respiratory systems in mice after an intratracheal injection of 50 *μ*g. The animals were sacrificed at 24 hours after this exposure, then, a broncho-alveolar lavage was performed, and the cells were stained with Diff-Quick. The results on cells are found in Figure 1.

There was an infiltration of neutrophils, eosinophils, and mononuclear cells, which was concomitant with an increased local expression of proinflammatory cytokines such as tumor necrosis factor-*α*, interleukin (IL)-1*β*, IL-6, as well as chemokines such as macrophage inflammatory protein-1*α*, macrophage chemoattractant protein-1, normal T cells and their secretion, and eotaxin. The lung inflammation with enhanced expression of proinflammatory proteins caused by CSBG was directly related to its structure, since structurally degraded products of CSBG by formic acid induced negligible responses in the lung. Taken together, these data suggest that pulmonary exposure to CSBG in the form and the dose applied here can induce lung inflammation through enhanced expressions of proinflammatory cytokines and chemokines. In contrast to these results, other studies have not found an inflammatory response neither in animals [4] nor in humans [5]. This discrepancy is probably due to differences in dose and solubility of the 1,3-*β*-glucans used and/or the exposure modes (intratracheal administration versus inhalation exposure). It illustrates the complexity in the effects of different glucans and further studies need to be undertaken to relate the different cellular reactions to risk for diseases.

## Figures and Tables

**Figure 1:** Cells in lavage fluid after intratracheal instillation of suspension fluid (vehicle), 1,3-*β*-glucan (CSBG), and degraded CSBG (DEG-CSBG). *n* = 8–12 in each group. Data are mean ± SEM. *P > 0.05 versus other treatment.

## References

[1] Raetz CRH (1990). Biochemistry of endotoxins. *Annual Review of Biochemistry*.

[2] Kimpe SJD, Kengatharan M, Thiemermann C, Vane JR (1995). The cell wall components peptidoglycan and lipoteichoic acid from *Staphylococcus aureus* act in synergy to cause shock and multiple organ failure. *Proceedings of the National Academy of Sciences of the United States of America*.

[3] Ohno N, Uchiyama M, Tsuzuki A (1999). Solubilization of yeast cell-wall *β* − (1 → 3)-D-glucan by sodium hypochlorite oxidation and dimethyl sulfoxide extraction. *Carbohydrate Research*.

[4] Korpi A, Kasanen J-P, Kosma V-M, Rylander R, Pasanen A-L (2003). Slight respiratory irritation but not inflammation in mice exposed to (1 → 3)-*β*-D-glucan aerosols. *Mediators of Inflammation*.

[5] Thorn J, Beijer L, Rylander R (2001). Effects after inhalation of (1 → 3)-*β*-D-glucan in healthy humans. *Mediators of Inflammation*.

# Abstract: INNATE IMMUNE RESPONSE TO FUNGAL *β*-GLUCANS VIA THE SPECIFIC CELL SURFACE RECEPTOR DECTIN-1

**Authors:** Yoshiyuki Adachi

**Affiliations:** Laboratory for Immunopharmacology and Microbial Products, Tokyo University of Pharmacy and Life Sciences, 1432-1 Horinouchi, Hachioji, Tokyo 192-0392, Japan

## INTRODUCTION

Dectin-1 is a specific receptor for 1,3-*β*-glucans which is expressed on various kinds of leukocytes, such as macrophages, neutrophils, and dendritic cells. It plays an important role for activating innate immune response to fungal infection and functions as a signaling receptor for the activation of superoxide production and inflammatory gene expression by the NF-*κ*B-activation pathway.

There is a need to further characterize the stimulatory 1,3-*β*-glucan agonists for Dectin-1 in view of the diversity of the various physicochemical properties of 1,3-*β*-glucans such as branching ratio, length of branched glucosyl residues, degree of polymerization of main glucosyl chain and solubility. In terms of branching structure 1,3-*β*-glucans may be classified into three major groups: (1) a monoglucosyl branched linear 1,3-*β*-glucan as represented by Sonifilan (SPG), a soluble glucan obtained from liquid-cultured extracellular polysaccharide of *Schizophyllum commune*. (2) Tree-like branched main 1,3-glucosyl linkage with 1,6-monoglucosyl branched 1,3-*β*-glucans as represented by Lentinan and SCG, which are extracted from the fruit body of edible mushrooms. (3) a linear 1,3-*β*-glucan with various lengths of the 1,6-glucosyl chain as represented by CSBG and OX-CA from yeast (*Candida albicans* ) or Zymosan (*Saccharomyces cerevisiae*).

In this study, we used several structurally defined and physicochemical different 1,3-*β*-glucans and evaluated innate immune responses such as NF-*κ*B activation and cytokine production from Dectin-1-expressing cells. 

## RESULTS AND DISCUSSION

**Molecular mechanisms for 1,3-*β*-glucan recognition and NF-*κ*B activation in Dectin-1 transfectant**

Zymosan has been used as a model compound of yeast microorganisms and stimulates macrophages mainly through TLR2- and Dectin-1-mediated recognition. In order to examine the contribution of Dectin-1 in TLR2/MyD88-mediated NF-*κ*B activation, various Dectin-1 mutants were tested in TLR2 coexpressed HE293 transfectant. The NF-*κ*B activation in TLR2 transfectant was significantly enhanced by coexpression of wild-type Dectin-1. The Dectin-1 mutants, in which amino acid residues of extracellular Trp221 and His223 (carbohydrate recognition domain) or intracellular Tyr15 (ITAM motif) are replaced with alanine or phenylalanine, had a lower activation of NF-*κ*B. This is comparable to TLR2-alone-expressed HEK293 [1]. However, wild-type Dectin-1 transfectant without TLR2 did not respond to stimulation with Zymosan. In the case of stimulation of Dectin-1/TLR2 coexpressed HEK293 transfectant with purified particulate 1,3-*β*-glucan (OX-CA), the cell showed no activation of NF-*κ*B.

These results indicate that Dectin-1 functions as a costimulatory signaling receptor for TLR2-mediated NF-*κ*B activation in HEK293, and Zymosan may have multiple agonist effects, not only as a *β*-glucan, but also involving TLR2 reactive ligands.

**Structure-activity relationship on Dectin-1/Card9-mediated NF-*κ*B activation**

Card9 is newly identified signaling molecule in innate immune response and mediates Dectin-1-related NF-*κ*B activation. To examine which type of 1,3-*β*-glucan can stimulate the Dectin-1/Card9/Bcl10 signaling pathway, various 1,3-*β*-glucans were tested, using Dectin-1/Card9/Bcl10 expressing HEK293T transfectant. Incubation of the cells with Zymosan and OX-CA showed enhanced NF-*κ*B activation, but CSBG (the soluble form of OX-CA), SCG, and SPG did not increase the NF-*κ*B-assisted luciferase expression [2]. These results suggest that the particulate form of 1,3-*β*-glucan stimulates Dectin-1/Card9/Bcl10 signaling, and that this activation pathway does not require TLR2-reactive ligands.

**Innate immune response to soluble 1,3-*β*-glucans in Dectin-1 knockout mice**

Although the above results suggest that only particulate 1,3-*β*-glucans are active on the Dectin-1-mediated immune response, it has been demonstrated that a soluble 1,3-*β*-glucan is immune stimulatory to leukocytes [3]. To investigate the Dectin-1-mediated signaling of soluble 1,3-*β*-glucan, bone marrow-derived dendritic cells (BMDC) from dectin-1^−/−^ mice were used in a cytokine production assay [4]. TNF-*α* production from dectin-1^+/+^-derived BMDC was increased by coincubation with OX-CA or SCG, but the cells from dectin-1^−/−^ mice did not show any significant production in response to the 1,3-*β*-glucans. On the contrary, the lipopolysaccharide-induced TNF-*α* production by dectin-1^−/−^ BMDC was similar to that of dectin-1^+/+^. These results suggest that the immune response to soluble 1,3-*β*-glucan also requires the Dectin-1 molecule for cell activation.

## CONCLUSION

Dectin-1 plays an important role for leukocyte activation in response to particulate 1,3-*β*-glucans and also to soluble forms with tree-like branched 1,3- and 1,6-*β*-glucosyl linkage. The Dectin-1-mediated activation signaling induced by such soluble 1,3-*β*-glucans may require other signaling molecules which are defect in HEK293 and which are able to support the known signaling molecules including Syk, Card9, and Bcl10.

## References

[1] Adachi Y, Ishii T, Ikeda Y (2004). Characterization of *β*-glucan recognition site on C-type lectin, dectin 1. *Infection and Immunity*.

[2] Ikeda Y, Adachi Y, Ishii T, Miura N, Ohno N Dissociation of TLR2-mediated innate immune response to Zymosan by the organic solvent-treatment without loss of dectin-1 reactivity.

[3] Harada T, Kawaminami H, Miura NN (2006). Mechanism of enhanced hematopoietic response by soluble *β*-glucan SCG in cyclophosphamide-treated mice. *Microbiology and Immunology*.

[4] Saijo S, Fujikado N, Furuta T (2007). Dectin-1 is required for host defense against *Pneumocystis carinii* but not against *Candida albicans*. *Nature Immunology*.

# Abstract: EFFECTS OF INHALED 1,3-*β*-GLUCAN: PROPOSAL FOR A MODEL

**Authors:** Ragnar Rylander

**Affiliations:** BioFact Environmental Health Research Center, Bjorkasv 21, 44391 Lerum, Sweden

## INTRODUCTION

It has been shown above that 1,3-*β*-glucans are present in many general and occupational environments and that they induce cell activation through receptors such as Dectin-1. A risk assessment of exposure to 1,3-*β*-glucans in the environment is complicated by the simultaneous presence of a variety of other agents including microbial cell wall agents such as bacterial endotoxin. An understanding of the biological effects of 1,3-*β*-glucan must thus be based mainly on information derived from experiments with the pure substance. In the following an attempt is made to synthesize the available information on the toxicity of 1,3-*β*-glucan with the goal to propose a model for the reactions after inhalation at exposure levels similar to those encountered in occupational and general environments.

## EXPERIMENTAL EVIDENCE

The effects of purified 1,3-*β*-glucan have been studied in a number of studies on animals and humans as previously reported [1]. A possible general error in such studies is that the extraction of 1,3-*β*-glucan from the fungal cell wall might affect its chemical characteristics and hence the biological effect. It is also important to recognize that any effect studied is dependent on the dose and that the dose-response is not always linear but might have a U-form (Hormesis dose-response). The absence of an effect at a low dose thus does not contradict the possibility that it may occur at a higher dose. This might explain some of the conflicting data in the literature regarding the stimulation of inflammatory cytokines by 1,3-*β*-glucan. In inhalation experiments with doses similar to those found in the environment, there is no evidence for an inflammatory response [2]. At high dose levels, however, and using water soluble forms of 1,3-*β*-glucan, an inflammation can be induced and cytokine stimulation has been reported in in vitro experiments (see reviews in [1, 3]).

It has been demonstrated that 1,3-*β*-glucan influences the immune system. The leukocyte invasion into the airways induced by an acute inhalation of endotoxin is blunted by a previous inhalation of 1,3-*β*-glucan [4]. The secretion of TNF*α* from peripheral blood monocytes was decreased after an acute inhalation of 1,3-*β*-glucan [5, 6]. A depression of the inflammatory response could also explain the prolonged survival seen in mice with polymicrobial sepsis, presumably by preventing the increase in transcription factor NF-*κ*B [7]. This blunting of the inflammatory response could be interpreted as beneficial but conceptually it represents an interference with normal defence mechanisms against inhaled agents particularly microbes. In chronic exposures the normal adaptation to endotoxin does not take place if 1,3-*β*-glucan is given simultaneously; another example of interference with defence mechanisms [8].

Other data suggest that 1,3-*β*-glucan supports the Th2-related maturation of lymphocytes, increases the number of eosinophils in the airways after long-term inhalation exposure [9], and potentiates an ovalbumin induced eosinophilia [6]. A study in mice demonstrated that 1,3-*β*-glucan treatment supported a Th2-dependent antibody response to ovalbumin, suggesting that 1,3-*β*-glucan alters the susceptibility to environmental antigens [10].

There is evidence for this effect of 1,3-*β*-glucan in population studies. An increased proportion of persons with atopic sensitization or total IgE has been reported from mouldy environments [11]. A relationship has been found between airborne viable spores and total IgE levels in children [12]. A relation between markers of atopy and exposure to moulds was found in several studies [13–15]. An increased incidence of atopic persons was found among persons living in houses with higher levels of 1,3-*β*-glucan [16]. One study reported an association between 1,3-*β*-glucan in floor dust and the amount of IgE and the proportion of blood eosinophils but not with cellular markers of inflammation [17]. Among children at risk for allergy, exposure to Aspergillus at home was related to a lower content of Th1 cytokines, such as Interferon *γ* (IFN*γ*), TNF*α*, and Interleukin-2 secreted from T-cells [18]. A previously described development of allergy in adults working in a mouldy daycare centre could also be explained by such a mechanism [19]. In a recent study on young children, an inverse relationship was found between home levels of 1,3-*β*-glucan and wheezing [20]. As wheezing in this age group is caused by inflammation due to viral infections, these results suggest a blunting of the inflammatory response by 1,3-*β*-glucan.

Several studies report an increased incidence of infection among school children in environments with mould with remediation after cleaning [21–23]. This supports a concept of a reduced resistance to pulmonary infections.

## THE MODEL

Based upon the material reviewed above, a model for the effects of 1,3-*β*-glucan after inhalation is depicted in Figure 2.

The body has a number of defence mechanisms against external challenges such as infectious, inflammagenic, and allergenic agents. It is suggested that a prolonged exposure to 1,3-*β*-glucan interferes with these defence mechanisms, leading to an increased risk for disease. If the effect of 1,3-*β*-glucan is a threshold phenomenon, there will not be a dose-response relationship with the exposure to moulds. Instead, the dose-response for the effective agent (e.g., endotoxin or allergen) in the environment will be shifted downwards and reflect sensitization. It is suggested that the model illustrated in Figure 2 could be used for further explorations on the effects of exposure to 1,3-*β*-glucan and moulds in the environment.

## Figures and Tables

**Figure 2:** Proposed model for 1,3-*β*-glucan activity after inhalation.

## References

[1] Young S-H, Castranova V (2005). *Toxicology of* 1 → 3*-Beta-Glucans: Glucans as a Marker for Fungal Exposure*.

[2] Rylander R, Young S-H, Castranova V (2005). (1 → 3)-*β*-D-glucan in the environment: a risk assessment. *Toxicology of* 1 → 3*-Beta-Glucans: Glucans as a Marker for Fungal Exposure*.

[3] Williams DL, Lowman DW, Ensley HE, Young S-H, Castranova V (2005). Introduction to the chemistry and immunobiology of *β*-glucans. *Toxicology of* 1 → 3*-Beta-Glucans: Glucans as a Marker for Fungal Exposure*.

[4] Fogelmark B, Goto H, Yuasa K, Marchat B, Rylander R (1992). Acute pulmonary toxicity of inhaled (1 → 3)-*β*-D-glucan and endotoxin. *Agents and Actions*.

[5] Beijer L, Thorn J, Rylander R (2002). Effects after inhalation of (1 → 3)-*β*-D-glucan and relation to mould exposure in the home. *Mediators of Inflammation*.

[6] Wan G-H, Li C-S, Guo S-P, Rylander R, Lin R-H (1999). An airbone mold-derived product, *β*-1,3-D-glucan, potentiates airway allergic responses. *European Journal of Immunology*.

[7] Williams DL, Ha T, Li C, Kalbfleisch JH, Laffan J, Ferguson DA (1999). Inhibiting early activation of tissue nuclear factor-*κ*B and nuclear factor interleukin 6 with (1 → 3)-*β*-D-glucan increases long-term survival in polymicrobial sepsis. *Surgery*.

[8] Fogelmark B, Sjöstrand M, Rylander R (1994). Pulmonary inflammation induced by repeated inhalations of *β*(1,3)-D-glucan and endotoxin. *International Journal of Experimental Pathology*.

[9] Fogelmark B, Thorn J, Rylander R (2001). Inhalation of (1 → 3) − *β*-D-glucan causes airway eosinophilia. *Mediators of Inflammation*.

[10] Ormstad H, Hetland G, Young S-H, Castranova V (2005). Adjuvant effects of *β*-glucan in a mouse model for allergy. *Toxicology of* 1 → 3*-Beta-Glucans: Glucans as a Marker for Fungal Exposure*.

[11] Stark PC, Burge HA, Ryan LM, Milton DK, Gold DR (2003). Fungal levels in the home and lower respiratory tract illnesses in the first year of life. *American Journal of Respiratory and Critical Care Medicine*.

[12] Su H-J, Wu P-C, Lei H-Y, Wang J-Y (2005). Domestic exposure to fungi and total serum IgE levels in asthmatic children. *Mediators of Inflammation*.

[13] Savilahti R, Uitti J, Roto P, Laippala P, Husman T (2001). Increased prevalence of atopy among children exposed to mold in a school building. *Allergy*.

[14] Schäfer T, Krämer U, Dockery D, Vieluf D, Behrendt H, Ring J (1999). What makes a child allergic? Analysis of risk factors for allergic sensitization in preschool children from East and West Germany. *Allergy and Asthma Proceedings*.

[15] Garrett MH, Rayment PR, Hooper MA, Abramson MJ, Hooper BM (1998). Indoor airborne fungal spores, house dampness and associations with environmental factors and respiratory health in children. *Clinical and Experimental Allergy*.

[16] Thorn J, Rylander R (1998). Airways inflammation and glucan in a rowhouse area. *American Journal of Respiratory and Critical Care Medicine*.

[17] Rylander R, Andersson B Microbial cell wall agents indoors in relation to some markers of inflammation.

[18] Müller A, Lehmann I, Seiffart A (2002). Increased incidence of allergic sensitisation and respiratory diseases due to mould exposure: results of the Leipzig Allergy Risk children Study (LARS). *International Journal of Hygiene and Environmental Health*.

[19] Rylander R (1997). Airborne (1 → 3)-*β*-D-glucan and airway disease in a day-care center before and after renovation. *Archives of Environmental Health*.

[20] Iossifova YY, Reponen T, Bernstein DI (2007). House dust (1 → 3)-*β*-D-glucan and wheezing in infants. *Allergy*.

[21] Pirhonen I, Nevalainen A, Husman T, Pekkanen J (1996). Home dampness, moulds and their influence on respiratory infections and symptoms in adults in Finland. *European Respiratory Journal*.

[22] Taskinen T, Hyvärinen A, Meklin T, Husman T, Nevalainen A, Korppi M (1999). Asthma and respiratory infections in school children with special reference to moisture and mold problems in the school. *Acta Paediatrica*.

[23] Savilahti R, Uitti J, Laippala P, Husman T, Roto P (2000). Respiratory morbidity among children following renovation of a water-damaged school. *Archives of Environmental Health*.

# Abstract: ROLE OF BETA-GLUCAN ANTIBODY IN FUNGAL INFECTIOUS DISEASES

**Authors:** K. Ishibashi, M. Motoi, M. Yoshida, N. N. Miura, Y. Adachi, N. Ohno

**Affiliations:** ^1^Laboratory for Immunopharmacology of Microbial Products, School of Pharmacy, Tokyo University of Pharmacy and Life Sciences, 1432-1 Horinouchi, Hachioji, Tokyo 192-0392, Japan; ^2^Division of Nephrology, Hachiouji Medical Center, Tokyo Medical University, 1163 Tatemachi, Hachioji, Tokyo 193-0998, Japan

## INTRODUCTION

The major components of the fungal cell wall are polysaccharides such as mannan, *β*-glucan, and chitin. *β*-glucan is the major component, accounting for approximately 50% of the cell wall mass [1, 2]. It is basically insoluble in water and alkaline solutions. In fungal infection diagnosis, serological and molecular tests are used to detect the fungus antigen, antibody, or gene. These tests are important in the early diagnosis as a supplement to the clinical and mycological diagnosis. The Limulus test using the 1,3-*β*-glucan-sensitive factor G is useful for the screening of general fungal infections [3]. The measured values correlate with clinical symptoms and pathological changes [4, 5].

Many researchers have examined the fungi-host relationship and antifungal protective immune mechanism. The importance of innate immunity (i.e., macrophages, neutrophils) and cellular immunity (T-helper type 1 cells) in the defense against fungi is widely acknowledged [6, 7]. Little is known about the effects of fungal antibody on the host response to fungal infection, and few fungal antigens have been characterized for their ability to elicit the production of protective antibodies.

In this study, we examined the reactivity of the anti-*β*-glucan antibody (anti-BGAb), the *β*-glucan-anti-BGAb interaction in vivo and in vitro, and the role of this antibody in fungal infections.

## RESULTS AND DISCUSSION

The measurements of anti-BGAb titers of sera were performed using an ELISA test, precoating with glucan preparations derived from various fungi. Binding of immunoglobulin was detected by peroxidase-conjugated antibody against each immunoglobulin isotype. The titer and reactivity of the anti-BGAb in healthy human volunteers were examined. Five kinds of polyclonal human immune globulin preparations were also tested.

All sera and globulins tested contained anti-BGAb. There was a tenfold or higher variation in anti-BG titer between values from different individuals but there was no significant difference between sampling occasions. Human normal immune globulin showed higher reactivity to *Candida* solubilized BG (CSBG), a long *β*-1,6-linked glucan chain attached to the *β*-1,3-glucan chain from *Candida albicans* [8] and Aspergillus solubilized BG (ASBG), mainly composed of *β*-1,3-glucan from *Aspergillus niger* [9]. There was only a weak immune globulin response to GRN and 6-branched *β*-1,3-glucan from the mushroom *Grifola frondosa* [10] (Figure 3).

The lower levels in subjects with infection may be caused by attachment of the antibodies to cell wall *β*-glucan, similar to the findings in mice where administration of whole *Candida* cells caused a gradual decrease in antibody titers [11]. The decrease in anti-BGAb titer could thus be related to the progression of a fungal infection in patients with mycosis.

Deep mycosis is a clinical problem also in animals. We compared the titer and reactivity of anti-BGAb among several animal species (monkey, horse, bovine, goat, pig, rabbit, mouse, dog, turkey, chicken) by ELISA, using plates precoated with various standard *β*-glucans or polysaccharides. Although species differences were present, anti-BGAb was found in every species and showed high reactivity to glucan derived from pathogenic fungal cell wall. Furthermore, we compared the anti-BGAb titer among fetal calf, calf and bovine. Fetal calf sera had very low titers but they were higher in calf and bovine sera. Also the titer was higher in the bovine sera than calf sera. These results suggest that anti-BGAb in native bovine sera is induced by fungi in the environment.

To assess if anti-BGAb could act on *Candida* cells, we measured its effect on the candidacidal activity of human macrophages derived from THP-1. *Candida albicans* cells, anti-BGAb or control were added to the THP-1 and incubated for 3 hours. After incubation, nonphagocytosed *Candida* cells were collected and placed on YPG agar. anti-BGAb enhanced the candidacidal activity which suggests that it may modify the host defense against *Candida* through opsonization.

We also examined the anti-BGAb interaction in vivo after administration of glucan. The anti-BGAb titer of DBA/2 mice which had received an intravenous dose of CSBG or ASBG decreased in a dose-dependent manner as compared to the titer before administration. These results suggest that anti-BGAb interacted with pathogenic fungal cell wall glucan specifically in vivo and that it was eliminated from the blood as an antigen-antibody complex.

In a case study, anti-BGAb titers were measured in patients with aspergillosis (*n* = 2) and carinii pneumonia (*n* = 3) and with 1,3-*β*-glucan positive sera. The average titer in normals was 2371 units/mL, compared to 414 units/mL among those with fungal infection. The patients had significantly lower titers compared with normal volunteers. The anti-BGAb titer was reversely related to the clinical symptoms and galactomannan.

It has previously been reported that plasma levels of 1,3-*β*-glucan are elevated in dialysis patients [12]. We compared the anti-BGAb titer of dialysis patients and healthy volunteers. In dialysis patients, the titer was lower than in healthy volunteers. Long-term (15–40 years) dialysis patients had lower titers than short-term (<5 years) dialysis patients.

In conclusion, available data suggest that anti-BGAb could play a role for *β*-glucan recognition and induce clearance of pathogenic fungi and biological activity through collaboration with other recognition molecules such as *β*-glucan receptor or complement in human. Anti-BGAb interacted with pathogenic fungal cell wall glucan in vivo and was eliminated from the blood as an antigen-antibody complex. Measurements of anti-BGAb could be useful as a response index of pathogenic fungal cell wall *β*-glucan.

## Figures and Tables

**Figure 3:** Comparison of reactivity of human immunoglobulin (polyglobin *N* × 1000) to various antigen-coated plates. CSBG = *Candida* solubilized cell wall *β*-glucan, ASBG = Aspergillus solubilized cell wall *β*-glucan , GRN = Grifolan from G. frondosa, LPS = lipopolysaccharide.

## References

[1] Chaffin WL, Lopez-Ribot JL, Casanova M, Gozalbo D, Martinez JP (1998). Cell wall and secreted proteins of *Candida albicans*: identification, function, and expression. *Microbiology and Molecular Biology Reviews*.

[2] Ruiz-Herrera J (1967). Chemical components of the cell wall of *Aspergillus* species. *Archives of Biochemistry and Biophysics*.

[3] Obayashi T, Yoshida M, Mori T (1995). Plasma (1 → 3)-*β*-D-glucan measurement in diagnosis of invasive deep mycosis and fungal febrile episodes. *Lancet*.

[4] Yoshida M, Obayashi T, Iwama A (1997). Detection of plasma (1 → 3)-*β*-D-glucan in patients with fusorium, trichosporon, saccharomyces and acremonium fungaemias. *Journal of Medical and Veterinary Mycology*.

[5] Yuasa K, Goto H, Iguchi M, Okamura T, Ieki R (1996). Evaluation of the diagnostic value of the measurement of (1 → 3)-*β*-D-glucan in patients with pulmonary aspergillosis. *Respiration*.

[6] Bistoni F, Vecchiarelli A, Cenci E, Puccetti P, Marconi P, Cassone A (1986). Evidence for macrophage-mediated protection against lethal *Candida albicans* infection. *Infection and Immunity*.

[7] Romani L, Mencacci A, Cenci E (1992). Course of primary candidiasis in T cell-depleted mice infected with attenuated variant cells. *Journal of Infectious Diseases*.

[8] Ohno N, Uchiyama M, Tsuzuki A (1999). Solubilization of yeast cell-wall *β* − (1 → 3)-D-glucan by sodium hypochlorite oxidation and dimethyl sulfoxide extraction. *Carbohydrate Research*.

[9] Ishibashi K, Miura NN, Adachi Y, Tamura H, Tanaka S, Ohno N (2004). The solubilization and biological activities of *Aspergillus*
*β* − (1 → 3)-D-glucan. *FEMS Immunology and Medical Microbiology*.

[10] Ohno N, Adachi Y, Suzuki I (1986). Antitumor activity of a *β*-1,3-glucan obtained from liquid cultured mycelium of Grifola frondosa. *Journal of Pharmacobio-Dynamics*.

[11] Ishibashi K, Yoshida M, Nakabayashi I (2005). Role of anti-*β*-glucan antibody in host defense against fungi. *FEMS Immunology and Medical Microbiology*.

[12] Kato A, Takita T, Furuhashi M, Takahashi T, Maruyama Y, Hishida A (2001). Elevation of blood (1 → 3)-*β*-D-glucan concentrations in hemodialysis patients. *Nephron*.

# Abstract: AUTOPSY-BASED REAPPRAISAL OF THE SERUM *β*-GLUCAN ASSAY FOR THE DIAGNOSIS OF INVASIVE FUNGAL INFECTION

**Authors:** Taminori Obayashi, Nobuaki Funata

**Affiliations:** Medical Laboratory and Pathology, Tokyo Metropolitan Cancer and Infectious Diseases Center, Komagome Hospital, Honkomagome 3-18-22, Bunkyo-ku, Tokyo 113-8677, Japan

## PURPOSE

To reexamine the efficacy of serum *β*-glucan assay in the diagnosis of invasive fungal infection.

##  METHODS 

A total of 456 autopsy records consecutive over the 6-year period from January, 2000 to December, 2005 were reviewed for the presence or absence of invasive fungal infection. The sensitivity of the assay was calculated based on those cases that were pathologically proved to be invasive fungal infections and had been tested for serum *β*-glucan within 2 weeks before death. Likewise the sensitivity of blood culture was obtained for comparison. The specificity of the *β*-glucan assay was determined on those cases that had no evidence of invasive fungal infection and had had a glucan test within 2 weeks before death. The cutoff was set at 30 pg/mL, because the upper limit of the normal range was 25 pg/mL. The positive and negative predictive values of the test were estimated against cutoffs at 30, 60, and 80 pg/mL based on a model population whose prevalence of invasive fungal infection was simulated to that of the present series. In addition, the concordance between serum *β*-glucan assay and blood culture was studied by reviewing all the records of blood culture over the same 6-year period and collecting cases that grew fungi. Those who had been tested for serum *β*-glucan within 2 weeks of blood sampling for culture were selected and the concordance was calculated. 

## RESULTS

Out of 456 autopsies, 54 (12%) had invasive fungal infections. Leukemias were the most frequent underlying disease (52%), followed by lymphomas (13%) and AIDS (13%). The most frequent causative fungus was Aspergillus (70%). Out of the 54 cases with fungal infection, serum *β*-glucan was measured within 2 weeks before death in 41 cases, and 39 of those had a concentration higher than the cutoff value. One of the two negative cases was infected with Zygomycetes, a fungus that does not produce *β*-glucan. With this case excluded, the sensitivity of the serum *β*-glucan assay was 97.5% (39/40). Blood culture was performed in 48 of the 54 cases, and four of them were positive for fungi. The sensitivity of blood culture was thus only 8.3%. 

Out of 402 cases without fungal infections, 63 was tested for serum *β*-glucan within 2 weeks before death, and 54 of them showed the concentration lower than the cutoff. Thus, the specificity of *β*-glucan assay was 85.7%. The positive and negative predictive values of the *β*-glucan assay were 46.3% and 99.6% with cutoff at 30 pg/mL; 70.9% and 98.3% at 60 pg/mL, and 87.0% and 97.4% at 80 pg/mL. 

During the same 6 years, there were 21 fungus-positive blood cultures that were preceded or followed by serum *β*-glucan measurement within two weeks. The concentration of *β*-glucan was above 30 pg/mL in 17 cases. The concordance with blood culture, therefore, was 81% with the latter as a gold standard. 

## CONCLUSION

Serum *β*-glucan assay is a highly sensitive and highly specific test for invasive fungal infection. With a cutoff set at 80 pg/mL, the test will rule in or rule out the diagnosis of invasive fungal infection with about 90% probability in hospitals where many patients with haematological malignancies and cancers are cared for.

# Abstract: BACTERICIDAL AND LPS-NEUTRALIZING ACTIVITIES OF A HUMAN ANTIMICROBIAL CATHELICIDIN PEPTIDE CAP18/LL-37

**Authors:** Isao Nagaoka, Hiroshi Tamura, Michimasa Hirata

**Affiliations:** Department of Host Defense and Biochemical Research, School of Medicine, Juntendo University, 2-1-1 Hongo, Bunkyo-ku, Tokyo 113-8421, Japan

## INTRODUCTION

Peptide antibiotics exhibit potent antimicrobial activities against both Gram-positive and Gram-negative bacteria, fungi, and viruses, and they form one group of effector components in the innate host defense system. The peptide-based defense in mammals against invading microbes relies on the two evolutionally distinct groups of antimicrobial peptides, defensins, and cathelicidins, which have been identified in several epithelial tissues and in the granules of phagocytes. About 30 cathelicidin members have been identified from various mammalian species; however, only one cathelicidin hCAP18 (human cationic antibacterial protein of 18 kDa) has been found in humans, and its carboxyl terminal antibacterial peptide called LL-37 comprising 37 amino acid residues (L^1^LGDFFRKSKEKIGKEFKRIVQRIKDFLRNLVPRTES^37^) has been identified. Secondary-structure predictions indicate that LL-37 adopts an *α*-helical amphipathic conformation; the helical wheel regions are clearly amphipathic and subtended by the hydrophilic (positively charged) and hydrophobic sectors.

Among antibacterial peptides, defensins completely lose their antibacterial activities in the extracellular milieu containing a physiological concentration of NaCl (150 mM) and serum. In contrast, LL-37 can exhibit antibacterial activities against Gram-negative and Gram-positive bacteria under these conditions. Furthermore, LL-37 can bind to lipopolysaccharide (LPS) and neutralize its biological activities. Thus, LL-37 and its related derivatives could be attractive candidates for therapeutic agents that can be used not only for bacterial infections but also endotoxin shock/sepsis caused by Gram-negative bacterial infections.

A short fragment peptide (18-mer; K^15^ to V^32^) of LL-37 displayed an amphipathic *α*-helical structure as illustrated in Figure 4.

This fragment possessed antibacterial and LPS-neutralizing activities almost equal to those of the parent LL-37 peptide. To develop the cathelicidin-derived antimicrobial peptides with enhanced biological activities, we utilized the 18 mer peptide (K^15^-V^32^) of human cathelicidin LL-37 (L^1^-S^37^) as a template, and evaluated the activities of its peptide derivatives [1, 2]. By replacement of E^16^ and K^25^ with two L residues, hydrophobicity of the peptide was increased, and hydrophobic sector in the helix was extended (Figures 1 and 2; 18-mer K^15^-V^32^ versus 18-mer LL). Concomitantly, pI value was increased from 11.50 to 12.22. Furthermore, by replacement of Q^22^, D^26^,and N^30^ with three K residues, hydrophilicity of the peptide was enhanced, and positively charged hydrophilic sector in the helix was expanded (18-mer LL versus 18-mer LLKKK), accompanied with the further increase of pI value from 12.22 to 12.82.

## ANTIBACTERIAL ACTIVITIES 18-MER PEPTIDES

Antibacterial activities of the peptides were assessed by alamarBlue assay using *Staphylococcus aureus* (MSSA, meth- icillin-sensitive *Staphylococcus aureus*; MRSA methicillin-resistant *Staphylococcus aureus*), *Escherichia coli*, *Streptococcus pneumoniae*, *Streptococcus pyogenes*, and *Pseudomonas aeruginosa* as target organisms [1]. The 18-mer peptides as well as LL-37 inhibited the growth of these bacteria, and 18-mer LLKKK was the most potent among the peptide derivatives (Figure 5).

To further elucidate the mechanism for the antibacterial actions of LL-37-derived peptides, we assessed their membrane-permeabilization activities using *E. coli* ML-35p as a target organism. The peptide derivatives induced the outer and inner membrane permeabilization. The 18-mer LLKKK was the most potent among the LL-37-derived peptides at inducing the outer- and inner-membrane permeabilizations [1].

## LPS-NEUTRALIZING ACTIVITIES OF 18-MER PEPTIDES

Further, we evaluated the LPS-neutralizing activities of LL-37-derived peptides by examining their effects on the binding of FITC-conjugated LPS to CD14-positive cells using a murine macrophage cell line RAW 264.7 [2]. The18-mer peptides as well as LL-37 suppressed the binding of FITC-conjugated LPS to RAW 264.7 cells, and 18-mer LLKKK was the most potent among the peptides tested at inhibiting the binding of FITC-conjugated LPS to RAW 264.7 cells (Figure 5). We also revealed that the 18-mer K^15^-V^32^, 18-mer LL, and 18-mer LLKKK peptides have a potential to bind to LPS, and 18-mer LLKKK was the most potent at binding to LPS and inhibiting LPS-LBP interaction [2].

Using D-galactosamine-sensitized mice, we assessed the effects of 18-mer peptides on lethal LPS activity in vivo [2]. The administration of 18-mer K^15^-V^32^, 18-mer LL, 18-mer LLKKK, or LL-37 increased the survival rate, and 18-mer LLKKK administration was the most protective. In agreement with this, administration of an 18-mer peptide or LL-37 markedly lowered the LPS-induced increase in serum TNF-*α* levels, and 18-mer LLKKK had the largest effect.

## PERSPECTIVE

For prevention of bacterial infections, much attention has focused on the low-molecular-weight cationic antimicrobial peptides. Bacterial membrane contains large amounts of negatively charged phospholipids such as phosphatidylglycerol and cardiolipin. Thus, the cationic amphipathic antimicrobial peptides show high affinities for bacterial membrane components and kill bacteria by permeabilizing and/or disrupting bacterial membranes. Moreover, the positively charged amphipathic structures of antimicrobial peptides are assumed to be important for interaction with a negatively charged amphipathic LPS.

We have revealed that by modifying the hydrophobicity and cationicity of the amphipathic *α*-helical antimicrobial peptide (LL-37-derived 18-mer peptide), the in vitro and in vivo biological (antibacterial and LPS-neutralizing) activities of the peptide could be enhanced [1, 2]. On the other hand, it has been demonstrated that high concentrations of cationic antimicrobial peptides are occasionally toxic to host cells [3] and that cytotoxicity is correlated with the extent of the hydrophobic regions in the peptides [4]. Although many problems still need to be solved, antimicrobial peptides could become one of the new classes of antibiotics that can be used for treatment of bacterial infections and their related symptoms in the future.

## Figures and Tables

**Figure 4:** Helical wheel projections for LL-37 and its 18-mer peptide derivatives. Positively charged residues are in white circles, hydrophobic residues are in black circles, neutral hydrophilic residues are in gray circles, and negatively charged residues are boxed.

**Figure 5:** Hydrophilicity/hydrophobicity plots of LL-37 and its 18-mer peptide derivatives.

## References

[1] Nagaoka I, Kuwahara-Arai K, Tamura H, Hiramatsu K, Hirata M (2005). Augmentation of the bactericidal activities of human cathelicidin CAP18/LL-37-derived antimicrobial peptides by amino acid substitutions. *Inflammation Research*.

[2] Nagaoka I, Hirota S, Niyonsaba F (2002). Augmentation of the lipopolysaccharide-neutralizing activities of human cathelicidin CAP18/LL-37-derived antimicrobial peptides by replacement with hydrophobic and cationic amino acid residues. *Clinical and Diagnostic Laboratory Immunology*.

[3] Oren Z, Lerman JC, Gudmundsson GH, Agerberth B, Shai Y (1999). Structure and organization of the human antimicrobial peptide LL-37 in phospholipid membranes: relevance to the molecular basis for its non-cell-selective activity. *Biochemical Journal*.

[4] Tossi A, Sandri L, Giangaspero A (2000). Amphipathic, *α*-helical antimicrobial peptides. *Peptide Science*.

# Abstract: CONCLUDING REMARKS

**Authors:** Ragnar Rylander, Hajime Goto

**Affiliations:** ^1^BioFact Environmental Health Research Center, Bjorkasv 21, 44391 Lerum, Sweden; ^2^First Department of Internal Medicine, School of Medicine, Kyorin University, 6-20-2 Shinkawa, Mitaka-shi, Tokyo 181-8611, Japan

The knowledge on 1,3-*β*-glucan has increased tremendously since the first glucan workshop in 1990 [1] and a textbook is now available on the toxicity of different forms of 1,3-*β*-glucan after different modes of administration [2]. The understanding of the effects of 1,3-*β*-glucan is complicated by the many steric forms present and also by the simultaneous presence of other agents in environments where 1,3-*β*-glucans are present. Regarding the neutrophil-based acute inflammatory response it seems as if nonwater soluble 1,3-*β*-glucan cannot elicit this response whereas water soluble forms and high doses may do so. A major advance in the knowledge has been the identification of a specific receptor and the development of specific antibodies which will facilitate the estimates of exposure both in clinical cases and in the general population. Determinations of 1,3-*β*-glucan in serum have become established as a clinical tool in deep mycosis and work is underway to further evaluate the role of 1,3-*β*-glucan in pulmonary diseases. A major enigma is still the effect of 1,3-*β*-glucan on the immune system after chronic exposure and how this could relate to the risk for allergic sensitization and the suppressive effects on tumor growth seen in some early animal models. Hopefully the proceedings from this glucan workshop will not only point to the importance of 1,3-*β*-glucan in the clinical and general environment but also stimulate further research.

## References

[1] Rylander R, Goto H (1991). First Glucan Lung Toxicity Workshop. Committee on Organic Dusts Du sts ICOH, Report 1/91.

[2] Young S-H, Castranova V (2005). *Toxicology of* 1 → 3*-Beta-Glucans: Glucans as a Marker for Fungal Exposure*.
